# Is genotyping of single isolates sufficient for population structure analysis of *Pseudomonas aeruginosa* in cystic fibrosis airways?

**DOI:** 10.1186/s12864-016-2873-1

**Published:** 2016-08-09

**Authors:** Lea M. Sommer, Rasmus L. Marvig, Adela Luján, Anna Koza, Tacjana Pressler, Søren Molin, Helle K. Johansen

**Affiliations:** 1 The Technical University of Denmark, Center for Biosustainability, Hørsholm, Denmark; 2 Rigshospitalet, Department of Clinical Microbiology, Copenhagen, Denmark; 3 Rigshospitalet, Center for Genomic Medicine, Copenhagen, Denmark; 4 Exeter University, Cornwall, UK; 5 Rigshospitalet, Copenhagen CF Centre, Copenhagen, Denmark; 6 The Technical University of Denmark, Department of Systems Biology, Lyngby, Denmark

**Keywords:** Metagenomics, Longitudinal isolates, *Pseudomonas aeruginosa*, Cystic fibrosis

## Abstract

**Background:**

The primary cause of morbidity and mortality in cystic fibrosis (CF) patients is lung infection by *Pseudomonas aeruginosa*. Therefore much work has been done to understand the adaptation and evolution of *P. aeruginosa* in the CF lung. However, many of these studies have focused on longitudinally collected single isolates, and only few have included cross-sectional analyses of entire *P. aeruginosa* populations in sputum samples. To date only few studies have used the approach of metagenomic analysis for the purpose of investigating *P. aeruginosa* populations in CF airways.

**Results:**

We analysed five metagenomes together with longitudinally collected single isolates from four recently chronically infected CF patients. With this approach we were able to link the clone type and the majority of SNP profiles of the single isolates to that of the metagenome(s) for each individual patient.

**Conclusion:**

Based on our analysis we find that when having access to comprehensive collections of longitudinal single isolates it is possible to rediscover the genotypes of the single isolates in the metagenomic samples. This suggests that information gained from genome sequencing of comprehensive collections of single isolates is satisfactory for many investigations of adaptation and evolution of *P. aeruginosa* to the CF airways.

**Electronic supplementary material:**

The online version of this article (doi:10.1186/s12864-016-2873-1) contains supplementary material, which is available to authorized users.

## Background

Cystic fibrosis (CF) is a hereditary disease that causes malfunction of a chloride channel affecting the viscosity of the mucus on all muco-epithelial surfaces. Among other things, this results in impaired clearance of bacteria and other microorganisms from the airways with an associated increased risk of lung infections [1]. CF is the most common life-limiting genetic disorder in Caucasians, and lung infection with *Pseudomonas aeruginosa* is the primary cause of morbidity and mortality in CF patients [2, 3]. In the clinic, antibiotic treatment of these infections is usually based on the assumption that the bacterial populations in CF airways are homogeneous. In accordance with this assumption, several studies of the adaptation of *P. aeruginosa* to the CF airway environment with regard to e.g. resistance development [4, 5], metabolism [6], escape from the immune system [7], and transmission between niches in the airways of a patient [8] and between patients [9, 10], have primarily been carried out based on investigations of single longitudinally stored bacterial isolates [11–13].

However, it was recently shown that long-term bacterial infections of CF airways cannot solely be described as a “dominant lineage” model, where the infecting clone type adapts in a linear fashion, and new variants with increased fitness quickly outcompete their less fit ancestors [14]. Because of the heterogeneous environment of the CF airways, it is more likely a “diverse community” model that best describes the bacterial populations of the CF airways. This is a consequence of adaptive radiation and the development of different subpopulations with a high degree of polymorphic mutations [11, 14–16].

Thus, the question is whether genomic information from single isolates collected longitudinally from the same patient is sufficient for the characterization of adaptive and evolutionary processes in *P. aeruginosa* populations in CF airways. To answer this question, we have compared the sequences from longitudinally collected single isolates with single metagenomes from four CF patients. Therefore, rediscovery in the metagenomes of the genome sequences derived from the single isolates document that they constitute a substantial sub-population and thus are representative for the infecting population of the patient.

## Methods

We included four CF patients followed at the CF clinic at Rigshospitalet, Copenhagen, Denmark. The age of the patients ranged from 15 to 31 years and they were all recently diagnosed as chronically infected with *P. aeruginosa* (Copenhagen criteria [17]).

### Longitudinally collected single isolates

Genome sequenced longitudinally collected single isolates from the patients are described in details in Marvig et al. [13]. The single isolates included in this study cover *P. aeruginosa* sampled from: endolaryngeal suction, sputum samples, sinus samples taken at endoscopic sinus surgery, swabs from the sinuses, and bronchoalveolar lavage (BAL). Isolation and identification of *P. aeruginosa* from CF sputum samples was carried out as previously described [13].

### Metagenomic samples

Sputum samples were collected at the CF clinic at Rigshospitalet and samples were processed a median of 2 days after expectoration (range: 1–3 days, Additional file 1: Table S1). During the lag-time between expectoration and processing the samples were stored at 4 °C.

### Processing of metagenomic samples

The samples were treated with ca. 1:1 (v/v) 10× diluted Sputasol (Oxoid, c/o Thermo Fisher Scientific, UK) with continuous vigorous shaking for 30 min. for homogenisation.

The samples were divided into two fractions, one was plated on *Pseudomonas* isolation agar (PIA) plates and incubated in 24–72 h at 37 °C depending on when colonies appeared and before single colonies could no longer be picked. The single colonies were then grown in 96 well microtitter plates with 150 μl Luria Broth (LB) for 24–48 h. One hundred μl 50 % glycerol was added and the isolates were stored at −80°. The other fraction was directly subjected to DNA extraction (200–600 μl Sputasol treated sample), or stored at −20 °C until batch DNA purification could take place.

DNA extraction was carried out as in Lim et al. [18], with slight modifications: β-mercapoethanol was replaced by Sputasol treatment, centrifugation times were extended to 20 min at 3800×g, the volumes were adjusted to: 1.5 ml autoclaved milliQ, 100–200 μl DNase buffer and 3–6 μl DNase (depending on pellet size), and 1.5 SE buffer, with the Powersoil® DNA isolation kit (MO BIO Laboratories, USA) used according to the manufacturer, for DNA purification.

### Sequencing and analysis of metagenomic reads

Libraries were prepared in triplicates with Nextera XT Sample Preparation kit (Illumina Inc., USA) and pooled prior to sequencing on an Illumina MiSeq® bench-top sequencer with MiSeq reagent kit V2, 300 cycles (Illumina Inc., USA), resulting in 150 bp paired end reads. Initial analysis of the reads (also used for species identification) was carried out using Novoalign V2.07.18 (Novocraft Technologies [19]) for alignment to a library of human (GRCh37, ftp://ftp.ncbi.nlm.nih.gov/genomes/H_sapiens/Assembled_chromosomes/seq/hs_ref_GRCh37.p13_chr*mfa.gz), bacterial, archaeal, viral, and fungal (The NCBI database, downloaded: November 11^th^ 2013) sequences. All sequences aligning to the human genome were discarded.

The analysis of the *P. aeruginosa* population was performed according to Marvig et al. [13]. This implies: Alignment to the *P. aeruginosa* PAO1 reference genome (GenBank accession NC_002516.2; genome size of 6.4 Mb) with Bowtie 2 V2.0.2 [20] and The Genome Analysis Toolkit (GATK) V1.0.5083 [21] for realignment around indels. This simultaneously removed all non-*P. aeruginosa* reads from the metagenomic reads. The pileups of read alignments were performed with SAM tools V0.1.7 (r50) [22]. SNP calling from the metagenomic reads was carried out by manually identifying positions where mutations had previously been identified in the single isolates of the clone types from the same patients as the analysed metagenomes. The mutations of the single isolates have previously been discussed and presented in Marvig et al. [13]. Raw *de novo* assemblies of the metagenomes were carried out using Velvet [23] (version 1.2.10) with a k-mer length of 33 and the options set as follows: ‘-scaffolding no –ins_length 500 –cov_cutoff 3 –min_contig_lgth 500’. *De novo*-assembled genomes were aligned against each other using MUMmer3 [24] (version 3.23).

Maximum likelihood phylogenetic analysis was carried out with PAUP* [25] version 4.0b10 without root, using the alleles identified by the single isolate sequencing. The metagenomes were placed in the tree depending on their major alleles at positions where polymorphisms occurred. Maximum parsimonious phylogenetic analyses were also carried out with PAUP* version 4.0b10 using alleles of reference strain PAO1 as a root.

The rediscovery of SNPs from the single isolates in the metagenomes were done based on the assumption that if a SNP previously identified in the single isolates was present in more than 10 % of the reads, this was a rediscovered SNP. When looking at polymorphisms, only positions with a phred score >30 and with ≥4 read coverage were considered. This was then compared to the overall coverage of PAO1, to make the ratio presented in Fig. 6.

### Diversity measurement by phylogenetic analysis of single isolates

Diversity is shown as the mean distance to the Line of Decent (LOD) [12]. LOD is the immediate line from the root, here based on PAO1 as out-group, to the latest sampled isolate (the red line in the phylogenetic trees in Fig. 5), and the mean distance to LOD is the mean number of SNPs from this line to the remaining isolates.

### Diversity measurement of metagenomic samples

Polymorphic positions were identified as described above. Because of the varying coverage of the different samples, the polymorphisms found were compared to the overall coverage of the PAO1 genome, and diversity was calculated as a ratio of polymorphisms and coverage. The reason, for using polymorphisms as a diversity measurement, was based on the assumption that the more positions a population diverge in, the more diverse is it likely to be.

As example: if we have e.g. two or three subpopulations they will differ from each other in a number of positions creating more ambiguous base calls and thus a higher ratio of polymorphisms, than a single homogeneous population. However, it is not possible to differentiate between two, three, or more different subpopulations based on this method. This is because of the possibility of a deep phylogenetic branching of two subpopulations and a shallow branching of three or more subpopulations.

### Statistics

For comparisons of the rediscovery ratio of SNPs from single isolates in metagenomes and comparisons of diversity measurements of single isolates and metagenomes we used Fisher’s Exact Test with Holm correction for multiple testing, in R [26].

## Results

### Patient information and *P. aeruginosa* infection patterns

**Fig. 1:**
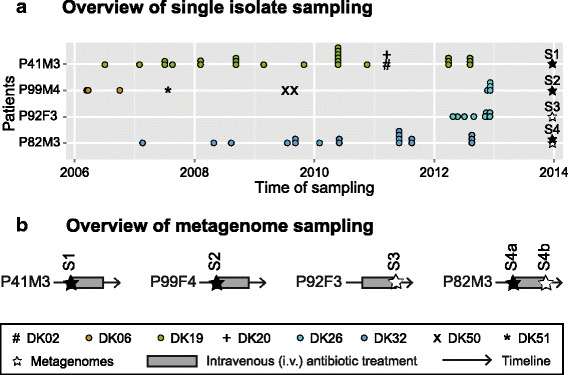
Overview. **a** Overview of single isolate sampling from four CF patients, clone types that are considered to be transient (found in 1–2 time points) are marked with #, +, x, or *, whereas clones considered to be persistent in the patient is marked by coloured circles. Metagenomes are marked with *stars*, *black* if sampled before i.v. antibiotic treatment, and *white* if sampled after i.v. antibiotic treatment. **b** Overview of metagenome sampling from patients, in correlation with 2-week i.v. antibiotic treatment

Four CF patients were enrolled in this study, median age 24 years; range 15–31, at the time of metagenome sampling. From each of the patients we have previously genome sequenced 9 to 27 longitudinally collected *P. aeruginosa* isolates covering 1–7 years of infection [13]. From the four patients we collected either one (*n* = 3) or two (*n* = 1) sputum samples for metagenomic analysis (Fig. 1a). Accordingly, sputum samples S1, S2, and S3 were sampled from patients P41M3, P99F4, and P92F3, respectively, and sputum samples S4a and S4b, separated by two weeks, were sampled from patient P82M3. The sputum samples used for the metagenome sequencing were collected approximately 1 year after the most recently genome sequenced single isolate. The time period between the most recently genome sequenced isolate and the metagenome is not critical, since the main question addressed here is whether or not the genotypes of the single isolates can be rediscovered in the metagenomic samples.

Three of the four patients (P41M3, P92F3, and P82M3) have infection patterns that are characteristic for the majority of the *P. aeruginosa* infected CF patients at the Copenhagen CF Center at Rigshospitalet [27], with a single primary clone type in the entire collection period. One patient (P99F4) has a change in clone type, where one clone type was outcompeted by another (Fig. 1a). All four patients in this study were recently diagnosed as chronically infected with *P. aeruginosa* according to the Copenhagen definitions at the time of metagenome sampling [17].

### Processing of sputum sample reads

The metagenome sequences were aligned to a database containing all bacterial, fungal, and viral genome sequences deposited at NCBI (see Methods). With a median of 96 % of all bacterial reads (Additional file 2: Table S2), *P. aeruginosa* was the dominating microbial species in the patients, corresponding to their clinical diagnosis as chronically infected with *P. aeruginosa*. We further aligned reads from the sputum metagenomes to the *P. aeruginosa* PAO1 reference genome, as we have previously done for the single isolates [13]. In all cases, the metagenomes had an average coverage of 5.99 Mbp (range: 5.90–6.04 Mbp) of the 6.3 Mbp PAO1 reference genome, by >3 reads and a phred score >30 (Additional file 3: Table S3). This high genomic coverage ensured that the presence or absence of polymorphisms in the metagenomes could be determined at the majority of genomic positions. On average, sequenced positions were covered by 10 to 31 reads giving us the opportunity to identify subpopulations that are present in more than 10 % of the population at the positions with the lowest coverage (Additional file 3: Table S3).

In order to compare the *P. aeruginosa* population structure and diversity as displayed by the single isolates and the compliance with the metagenomic read assemblies, we conducted a three step analysis: 1) Identification of the dominant clone type(s) in the sputum samples, 2) investigation if mutations in the genomes of the single isolates were also identified in the metagenomes, i.e. rediscovery of SNPs in the metagenomes, and 3) comparison of diversity measurements of the populations represented by the single isolates and the metagenomes.

### Identification of the dominant clone type(s)

To identify the *P. aeruginosa* clone types represented in the metagenomes, de novo assemblies of single isolates and metagenomes were compared. For each patient the clone types represented by the single isolates were compared with the metagenome(s) from the same patient.

For all four patients, the clone type of the most recently sampled single isolate corresponded to the clone type identified from the metagenome with less than 528 SNP of differences (median 131 SNPs, range 91–527 SNPs). In contrast, when comparing the metagenomes with single isolates of other clone types they differed by more than 16,268 SNPs (median 17,844 SNPs, range 16,269–30,918 SNPs) (Additional file 4: Table S4A and Table S4B).

This shows that for each patient the most recent clone type identified by the genome of the single isolate matches the dominating clone type in the *P. aeruginosa* population identified in the sputum sample metagenome.

### Rediscovery of SNPs in the metagenomes

Previous investigations of genome evolution in the clonal lineages of *P. aeruginosa* strains from each of the four patients [13] identified SNPs accumulating in the clonal populations. If these SNPs are indeed present in actual propagating lineages of the *P. aeruginosa* population of these patients, they should also be present in the metagenome(s). When looking at all the SNPs identified in all the single isolates, it is expected that the ratio of rediscovery of SNPs between single isolates and metagenomes from the same patients should exceed the ratio determined between single isolates and metagenomes of different patients. Further, this ratio should reach a value of one if all mutations found in the single isolates are also present in the metagenome.

**Fig. 2:**
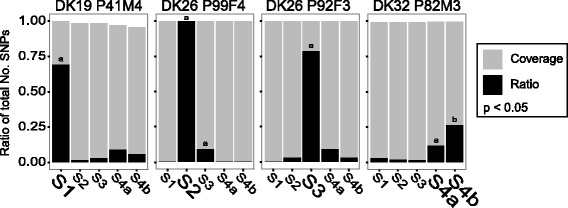
SNP rediscovery in metagenomes. Above each subfigure it is indicated which single isolates’ SNPs that have been sought rediscovered in the metagenomes (clone type and patient). The *grey bars* indicate the ratio of SNP positions that were sequenced in the metagenomes and the *black bars* indicate the ratio of the rediscovered reads to the sequenced positions. The metagenome(s) belonging to the same patient as the single isolates they are compared to is indicated with a larger font. NOTE, S2 and S3 are from different patients but the same clone type. *P* <0.05, Fisher’s exact test with Holm correction, significant differences are indicated by “a” and/or “b”

With the exception of patient P99F4 and P92F3, who are infected with the same clone type (DK26), the rediscovery of SNPs from the single isolates in the metagenome(s) of the same patient was found to be significantly higher than between patients (Fig. 2, *p* <0.05, Fisher’s exact test with Holm correction). This supports the specific linkage between single isolates and the *P. aeruginosa* population as a whole, as hypothesised above.

In one case (S2 from P99F4), the ratio of the rediscovery of SNPs reached one, suggesting that all SNPs identified in the single isolates are present in more than 10 % of the whole population. In all other cases the ratio was below one, which could be due to 1) not all mutations being fixed in the population, i.e. they were lost during the time of sampling of the single isolates (harbouring the mutations) until sampling of the metagenome, or 2) some of the mutations being present in only a small fraction (<10 %) of the population and therefore not sampled by the metagenomic reads. In the case of P92F3 the SNPs that were not rediscovered were only present in 11–22 % (Additional file 5: Table S5) of the single isolates, and thus could be explained by mutations not being fixed in the population.

The metagenomes S4a and S4b from patient P82M3 illustrate both explanations above: Firstly, the much lower ratio of rediscovery of SNPs in patient P82M3 compared to the other patients, may be explained by the presence of hypermutators in the *P. aeruginosa* population of P82M3. Hypermutators are known to accumulate many unfavourable mutations [28], which are not expected to remain in the population, thus leading to a low ratio of rediscovery (assuming that the mutations are not hitch-hiking with more favourable mutations). Secondly, the low coverage of the metagenomic samples (Additional file 3: Table S3) resulted in a higher percentage of the SNPs being rediscovered in the later metagenome (S4b) than in the early metagenome (S4a). The rediscovery of SNPs in the two metagenomes correspond to 26 % (122 of 461) and 12 % (54 of 461), respectively (Additional file 5: Table S5). This is contradictory since the mutations were previously identified in the single isolates and therefore must be present to some degree in S4a in order to be identified in S4b. This suggests that the subpopulation represented by the S4b metagenome is present below the limit of detection in the S4a metagenome sequences and is therefore not identified.

**Fig. 3:**
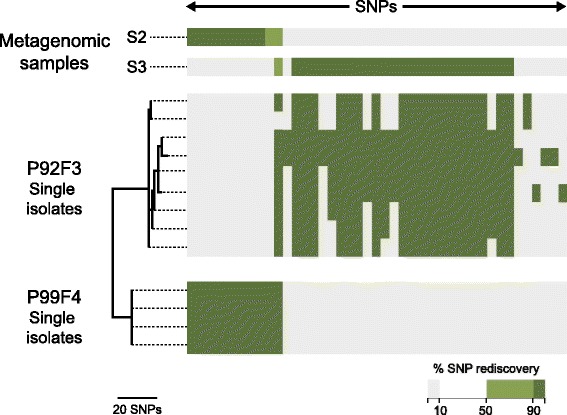
Patient specific correlation of rediscovered SNPs within a single clone type. A comparison of the SNPs found in the single isolates of the patients P99F4 and P92F3 as well as their respective metagenomes, S2 and S3. For the single isolates a *dark green* colour indicates the presence of SNPs and *white* the absence. For the metagenomes the percentage of reads covering the position of a SNP is indicated by *dark green* (>90 %), *light green* (51-90 %), or *white* (11-50 %) all considered to confirm the presence of the SNP in question. If the SNP is only found in <=10 % of the reads it is indicated by *grey* and not considered to be present in the metagenome(s)

For patients P99F4 and P92F3 the similar rediscovery ratios of SNPs between the metagenomes and the single isolates can be explained by a co-infection of the same clone type, DK26. This relationship was noted previously and seems to be the consequence of a patient-to-patient transmission event of the DK26 clone from P92F3 to P99F4 [13], explaining the lack of differentiation between the two *P. aeruginosa* populations. However, despite this close relationship between the populations, Fig. 3 shows that it is possible to distinguish between the SNPs of the single isolates and the respective metagenomes.

**Fig. 4:**
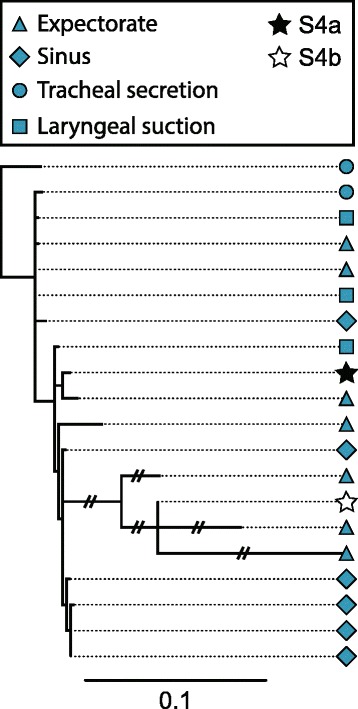
DK32 P82M3 maximum likelihood phylogeny including metagenomes. *Blue shapes* indicate single isolates sampled with different methods and from different locations (see legend) and *stars* indicate metagenomes. The *scale bar* indicates 0.1 likelihood of mutation

We have identified SNPs in genome sequences of longitudinal single isolates, which seem to be characteristic and representative for the patient community, including cases of infections caused by patient-to-patient transmitted clones. This patient specific relationship between metagenomes and single isolates is further documented by the phylogenetic analysis of the single isolates and metagenomes of the hypermutator population of patient P82M3 (Fig. 4), which shows that despite the highly increased mutation rate, the metagenomes are placed within the phylogeny of the single isolates from the patient (Fig. 4). This phylogeny also shows that the single isolates are not clustered depending on their origin of sampling, indicating that the population is mixed between the upper and lower airways and that the different subpopulations are not limited to a specific spatial position in the airways.

### Diversity of the *P. aeruginosa* populations

**Fig. 5:**
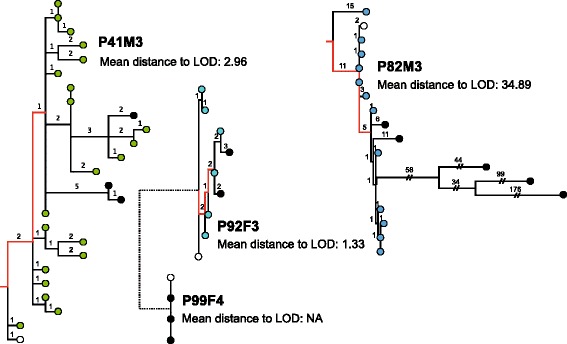
Mean distance to Line of Decent (LOD). Maximum parsimonious phylogenetic trees for all clone types identified in the metagenomes as being the latest. The *red line* in the trees indicates the LOD wherefrom SNPs (numbers on branches) have been counted. The LOD is set from the root (created for P41M3 and P82M3 using PAO1 as out-group) to the divergence of latest sampled isolates. *White circles* indicate the earliest sampled isolates and *black circles* indicates the latest sampled isolates from each patient, the coloured circles are comparable to the colours of Fig. 1. For each patient a mean distance to LOD is indicated below the patient name to the right of the corresponding tree. The mean distance to LOD of P82M3 is significantly different from the other patients, *p* <0.01, Fisher’s Exact test with Holm correction

**Fig. 6:**
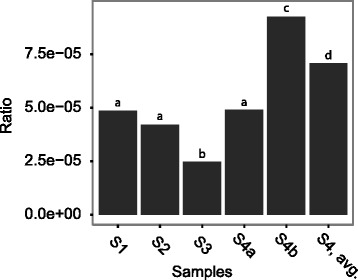
Polymorphic positions in the metagenomes. Because of the differing coverage of the PAO1 reference genome, the number of polymorphisms is shown as a ratio of polymorphisms and the coverage of each metagenome to PAO1. S4, avg. is the average of S4a and S4b. *p* <0.05 Fisher’s exact test with Holm correction

In the single isolates, the diversity of the *P. aeruginosa* populations was determined from the phylogenies as the mean distance to the Line of Decent (LOD) (Fig. 5). For the metagenome-estimated diversity (Fig. 6) we used the number of polymorphisms normalised to the number of positions covered in the PAO1 genome in order to correct for differences in coverage between the different metagenomes (see Methods for details). Because S4a and S4b (patient P82M3) are representative of the same population we chose to merge the samples to carry out the inter-patient comparison of diversity (Fig. 6: “S4, avg.”). In both the LOD calculations and the number of polymorphisms we find, that the hypermutator population of patient P82M3 had the highest diversity and that the patient with the shortest period of infection (P92F3), as expected, harboured the least diverse population, to some degree validating our method of diversity calculations. We calculated 34.89 and 1.33 mean distances to LOD for the two single isolate populations, and diversity ratios of 7.08E-05, and 4.20E-05 for the metagenome populations from the two patients P82M3 and P92F3, respectively. Thus, in both cases of diversity measurements both single isolates and metagenomes we saw a significant difference between the diversity of the *P. aeruginosa* populations of patient P82M3 and P92F3 (*p* <0.05, Fisher’s Exact test with Holm correction) (Figs. 5 and 6).

When analysing further the single population of patient P82M3, the diversity calculations for the samples S4a and S4b illustrate that exhaustive sampling is essential, not only when using single isolates but also for metagenomic samples, in order to get the true picture of the population diversity. Because these two metagenomes represent a non-mutator and a hyper-mutator subpopulation, respectively, they have significantly different diversity ratios (4.90E-05 and 9.25E-05, respectively, *p* <0.05 Fisher’s Exact test with Holm correction).

## Discussion

Airway infection in CF patients has attracted considerable interest as a model system for bacterial evolution and long-term human infections [29]. There is a number of reasons for this interest: 1) The infections are often mono-clonal lasting for decades, which can correspond to more than 100,000 bacterial generations [30], 2) sampling from the patients is relatively simple (sputum, BAL, suction), 3) the environmental conditions in the patient airways are very similar, and 4) isolate collections are found in many CF clinics covering long periods of sampling time. Several investigations of long-term CF airway infections based on the analysis of longitudinally collected single isolates of *P. aeruginosa* have been published in recent years [5, 6, 12, 13, 30–32], and some have also included cross-sectional analyses of the population diversity at the genomic level [11, 16, 33, 34]. One reason to question the validity of using single isolates to infer evolutionary dynamics of the entire population is the apparent heterogeneity of the *P. aeruginosa* population in the CF patients [11, 15, 16, 33–36].

In this study we have compared five meta-genomes obtained from four CF patient sputum samples with corresponding single, longitudinally collected, *P. aeruginosa* isolates, and a high degree of correlation was found within populations. The metagenomes were sampled from patients with the most common *P. aeruginosa* infection pattern, continuous culture of the same clone type, at the Copenhagen CF Centre [27], and they are therefore assumed to be representative for most of the CF patients and their lung infection. The collection of *P. aeruginosa* isolates from CF patients associated with the Copenhagen CF Clinic is comprehensive and characterized by frequent longitudinal sampling from the patients and frequent replicate isolates from individual patient samples. These features make the collection unique and useful for an assessment of the validity of single isolate analysis in relation to both biological and medical aspects. In general, single isolates and metagenome analyses depend on exhaustive sampling. Due to the possibility of temporal dominance by different subpopulations, as seen by the hyper-mutator population of P82M3, the metagenomic approach will also require multiple samples to reveal the profile and dynamics of *P. aerugonosa* populations. The results of this study, taken together with similar results from other studies [12], suggest that using comprehensive collections of longitudinally collected single isolates in the research of adaptation and evolution of *P. aeruginosa* to the CF airways will yield conclusive results.

One limitation of our study compared to e.g. that of Lieberman et al. [14] is the sequencing depth. This is especially true for the highly diverse population of P82M3, in which we were unable to identify subpopulations if present in less than 10 % of the population (the lowest coverage is 9.97). However, despite the lower sequencing depth, we were able to document a high degree of diversification of the populations in analogy with findings from Lieberman [14] and others [15, 16, 33]. In addition, we were also able to determine that the different subpopulations comprising this diversity differ in frequency over time. Especially in the hyper-mutator population, we noticed that the bacterial population is dominated by different subpopulations at different time points (Table 1 and Fig. 4).

**Table 1:** SNPs rediscovered in S4a and S4b

							Total no. of reads on position		% of reads with the mutation	
Position	Ref	Qry	Mutation (aa)	Mutation (bp)	PA no.	Gene name	S4a	S4b	S4a	S4b
5671	C	T	S466F	C1397T	PA0004	gyrB	15	13	7	100
2453983	G	A	G106D	G317A	PA2231	pslA	17	17	24	0
2640133	A	G	F43L	T127C	PA2386	pvdA	15	12	0	100
2926243	C	G	E64Q	G190C	PA2586	gacA	5	11	0	100
3970113	G	A	G172A	G58S	PA3545	algG	15	11	87	0
3971271	G	A	G1330A	G444S	PA3545	algG	19	13	0	100
5551035	A	C	V216G	T647G	PA4946	mutL	11	8	9	100
5677066	T	C	T265A	A793G	PA5040	pilQ	12	17	17	100
6028514	G	A	E325K	G973A	PA5361	phoR	5	12	100	0

Including examples of mutations not found in S4a but found in S4b (*Ref* reference base, *Qry* query base, *PA no.* PAO1 gene number, *aa* amino acid change, *bp* specific base pair substitution). NOTE: all mutation types are missense mutations

Population diversity was not the primary target of the investigations reported here due to the relatively short time frame of sampling and the resulting low number of mutations in the respective isolate genomes. However, the two cases of hyper-mutator isolates suggest that diversity is prevalent, resulting in significant population heterogeneity. This heterogeneity may be the result of spatial compartmentalisation of the CF airways and the confinement of different subpopulations to different niches [11, 35]. One obvious example of compartmentalization of the CF airways is illustrated by bacterial infections in both lungs and sinuses. Spatial isolation and adaptive radiation of different subpopulations in these niches has been suggested by Markussen [11] and Hansen [35]. In contrast, our current findings from longitudinally collected *P. aeruginosa* from upper and lower airways in younger CF patients [13] do not confirm this; instead, in accordance with Ciofu [37] and Johansen [38] we find that bacterial migration in both directions and consequential population mixing occurs between the upper and lower airways after a certain period of chronic infection (Fig. 4). Mixing of bacterial populations colonizing different airway compartments is also supported by other studies showing both genotypic and phenotypic overlap between samples from the upper and lower CF airways [37, 39].

Population mixing in CF airways is supported by frequent observations of clone type displacement; both in investigations of older CF patients with chronic *P. aeruginosa* lung infections [10] and in young patients with early colonization by *P. aeruginosa*. These findings are difficult to reconcile with spatial isolation and adaptive radiation, whether distribution of sub-populations is associated with the lung/sinus compartments or different sectors of the lungs as reported recently [40]. It is possible that the infections in fact switch between periods of adaptive radiation and spatial isolation of sub-populations resulting in diversity generation and periods of mixing caused by lung tissue changes. Such changes in population dynamics could explain the conflicting observations as well as the slow replacement of clone types, which sometimes take months or even years.

## Conclusions

We find a consistency between the genomic changes identified in the single isolates and in the metagenomes, which can only be explained by the propagation of mutations identified by the analysis of single isolates within the specific patient’s *P. aeruginosa* population. These findings underline the relevance of comprehensive longitudinal sampling of single isolates of *P. aeruginosa* for investigations of adaptation and evolution. We also find it equally important for the metagnomic approach to have comprehensive sampling, in order to provide valuable information about the *P. aeruginosa* population dynamics of CF patient airway infections. It is, however important to emphasise that the conclusions to be drawn from this type of investigation will not provide a complete picture of the population diversity in the respective samples.

## Abbreviations

BAL, bronchoalveolar lavage; CF, cystic fibrosis; DNA, deoxyribonucleic acid; LOD, line of decent; NCBI, national center for biotechnology information; *P. aeruginosa*, *Pseudomonas aeruginosa*; PIA, pseudomonas isolation agar; SNP, single nucleotide polymorphism

## Additional files

Additional file 1: Table S1. Sampling information of metagenomes. (XLSX 36 kb) Additional file 2: Table S2. Species ratios in metagenomes. All species presented have been detected in >0.5 % of the bacterial reads of at least one metagenomic sample, if a species is detected with <0.01 % it is shown here as 0.00 %. (XLSX 47 kb) Additional file 3: Table S3. Coverage of PAO1 by the metagenomes, coverage with >3 reads per position and phred score >30. (XLSX 37 kb) Additional file 4: Table S4A. Isolate and metagenome information of clone type (for the metagenomes this is the clone type that is supposed to be dominating, assumed from the single isolate information of the given patient). Table S4B: Genome comparisons of single isolates and metagenomes. (XLSX 42 kb) Additional file 5: Table S5. Detailed overview of SNPs found in single isolates and the rediscovery in the metagenomic reads. For each isolate it is indicated whether the mutation has been discovered or not, indicated by 1 and 0 respectively. For each metagenome the percentage of reads (%) representing the SNP found in the single isolates are shown together with the actual number of reads that have been called for each base, C, G, A, and T. For the metagenome(s) it is indicated at the bottom of the column the number of SNPs rediscovered in >10, 50 and 90 % of the metagenomic reads, as well as the total number of SNPs covered by the metagenomic reads. (XLSX 239 kb)
